# The role of the mycobiome in host physiology and disease: insights from rodent models

**DOI:** 10.1038/s41684-025-01620-6

**Published:** 2025-10-14

**Authors:** Karen Antunes, Nathaniel B. Willis, Joseph F. Pierre

**Affiliations:** Department of Nutritional Sciences, College of Agriculture and Life Science, University of Wisconsin−Madison, Madison, WI, USA

**Keywords:** Microbiology, Microbial communities, Fungi, Mucosal immunology

## Abstract

Mammals have evolved under an inseparable association with diverse microbial gut colonizers; thus, fine-tuned host−microbial interactions shape our susceptibility to immunological, oncogenic, metabolic and infectious outcomes. The past two decades have seen a rapid expansion of research into intestinal bacterial communities that impact host health in many preclinical and clinical settings. Meanwhile, other less abundant microorganism domains, including fungi, remain far less explored. This Review provides an overview of the literature on the gut mycobiome and evidence of its contributions to host physiology and disease over the past two decades in experimental rodent models, which have been foundational to our current understanding of host−microbe interactions.

## Abstract (web summary)

This Review examines the role of the gut mycobiome in health and disease states, based on findings from rodent models.

## Main

The mammalian gut is densely populated with trillions of microorganisms from multiple domains of life, including bacteria, fungi, eukaryotic and prokaryotic viruses, archaea and protozoans^1–4^. This complex mix of microorganisms has been termed the gut microbiome. The importance of the gut microbiome on host physiology is increasingly recognized, with research demonstrating microbial influence on digestion, neurological signaling, endocrine functions, drug absorption kinetics, immunological development and pathogen resistance^3,5^. Over the past two decades, research progress on gut bacteria and archaea, which share 16S ribosomal RNA, has grown exponentially following the advent of next-generation sequencing platforms; however, less focus has been placed on lower-abundance microorganisms such as yeasts and fungi comprising the gut mycobiome^4^. The dominant focus on gut bacteria is well rationalized given their abundant representation and the ease of sequencing the highly conserved 16S rRNA gene. Fungal DNA also contains a conserved nuclear ribosomal internal transcribed spacer that can be parallel amplified, similar to 16S rRNA. However, fungal cell walls are more difficult to digest during DNA isolation and the internal transcribed spacer gene varies in length between species^6^. Despite these limitations, the gut mycobiome has demonstrated some capacity to influence host health, disease etiology and pathogenesis (Table 1); however, evidence of its role in human physiology is still very limited.

**Table 1:** Summary of studies exploring fungal organisms and their morphology in association with the host as commensals, opportunistic pathogens or pathogens in relation to disease outcomes in rodent models

Fungal organism	Scientific classification (phylum, class)	Morphologies	Interaction with mammalian host	Disease relation
*Alternaria* spp.	Ascomycota, Dothideomycetes	Mold, hyphae	Commensal	−Increased abundance in the inflamed gastrointestinal tract^18^
*Aspergillus amstelodami*	Ascomycota, Eurotiomycetes	Mold, hyphae	Opportunistic pathogen	−Increased the severity of allergic airway disease^17^
*Aspergillus* spp.	Ascomycota, Eurotiomycetes	Mold-like, hyphae	Commensal, opportunistic pathogen, pathogen	−Enriched in diabetic rats^41^ −Increased after ethanol administration^46^ −Accelerated oncogenesis^51^
*Candida albicans*	Ascomycota, Saccharomycetes	Yeast and hyphae	Commensal, opportunistic pathogen	−Hepatocarcinogenesis^59^ −Protected against induced colitis^20^
*Candida glabrata*	Ascomycota, Saccharomycetes	Yeast or yeast-like	Opportunistic pathogen	−Worsening of induced colitis^22^
*Candida parapsilosis*	Ascomycota, Saccharomycetes	Yeast or yeast-like	Commensal, opportunistic pathogen	−Enhanced diet-induced obesity^36^
*Candida* spp.	Ascomycota, Saccharomycetes	Yeast or yeast-like	Commensal, opportunistic pathogen	−Increased abundance in the inflamed gastrointestinal tract^18^ −Delayed colitis healing process^29^ −Enriched in diabetic rats^41^
*Candida tropicalis*	Ascomycota, Saccharomycetes	Yeast or yeast-like	Commensal, opportunistic pathogen	−Reduced during antifungal treatment^17^ −Colorectal cancer^55^
*Cryptococcus* spp.	Basidiomycota, Tremellomycetes	Yeast or yeast-like	Pathogen	−Reduced abundance in the inflamed gastrointestinal tract^18^ −Enriched during metformin treatment^39^
*Dipodascaceae*	Ascomycota, Saccharomycetes	Yeast or yeast-like	Commensal	−Enriched during Alzheimer’s disease^63^
*Epicoccum nigrum*	Ascomycota, Dothideomycetes	Mold, hyphae	Commensal	−Increased severity of allergic airway disease^17^
*Fusarium* spp.	Ascomycota, Sordariomycetes	Hyphae	Commensal, opportunistic pathogen	−Enriched during metformin treatment^39^ −Increased after ethanol administration (alcoholic liver disease)^46^
*Humicola* spp.	Ascomycota, Sordariomycetes	Hyphae	Opportunistic pathogen	−Increased after ethanol administration (alcoholic liver disease)^46^
*Isaatchenkia* spp.	Ascomycota, Saccharomycetes	Yeast or yeast-like	Commensal	−Enriched in diabetic rats^41^
*Malassezia* spp.	Basidiomycota, Malasseziomycetes	Yeast-like	Commensal, opportunistic pathogen	−Accelerated oncogenesis^51^
*Meyerozyma guilliermondii*	Ascomycota, Saccharomycetes	Yeast or yeast-like	Commensal	−Increased after ethanol administration (alcoholic liver disease)^47^
*Naganishia globosa*	Basidiomycota, Tremellomycetes	Yeast-like	Opportunistic pathogen	−Enhanced diet-induced obesity^36^
*Penicillium* spp.	Ascomycota, Eurotiomycetes	Mold, hyphae	Commensal	−Reduced during antifungal treatment^17^
*Phialemonium* spp.	Ascomycota, Sordariomycetes	Hyphae	Opportunistic pathogen	−Reduced abundance in the inflamed gastrointestinal tract^18^
*Saccharomycetales* spp.	Ascomycota, Saccharomycetes	Yeast or yeast-like	Commensal	−Reduced abundance in the inflamed gastrointestinal tract^18^ −Decreased during metformin treatment^39^
*Saccharomyces cerevisiae*	Ascomycota, Saccharomycetes	Yeast or yeast-like	Commensal	−Protected against induced colitis^20^
*Saccharomyces cerevisiae* var. *boulardii*	Ascomycota, Saccharomycetes	Yeast or yeast-like	Commensal	−Reversed aspects of colitis^30^
*Tetrapisispora* spp.	Ascomycota, Saccharomycetes	Yeast	Commensal	−Enriched during metformin treatment^39^
*Thermothielavioides* spp.	Ascomycota, Sordariomycetes	Hyphae	Opportunistic pathogen	−Enriched during metformin treatment^39^
*Wallemia sebi*	Basidiomycota, Wallemiomycetes	Mold, hyphae	Commensal	−Increased severity of allergic airway disease^17^ −Reduced abundance in the inflamed gastrointestinal tract^18^
*Wickerhamomyces* spp.	Ascomycota, Saccharomycetes	Yeast or yeast-like	Commensal	−Increased abundance in the inflamed gastrointestinal tract^18^
*Wolfiporia cocos*	Basidiomycota, Agaricomycetes	Hyphae	Commensal	−Reduced hepatic stress after mycobiome disruption^47^

Given the complexity of the mycobiome, animal models have been extremely valuable for exploring host−microbe interactions, with the mouse being the most popular by far. Rodent models offer rapid reproduction and life cycles, cost effectiveness, genetic flexibility, human-relevant anatomical systems and control of environmental conditions allowing investigations under germ-free, gnotobiotic or specific pathogen-free conditions. Considering these factors, this Review provides an overview of the preclinical literature on the role of the gut mycobiota in host physiology and key disease states over the past two decades. The factors influencing gut colonization are also described.

## Factors influencing gut colonization

The gut mycobiome comprises 0.1% of the gut microbiome^7^. Like gut bacterial communities, fungal colonization begins at birth and is influenced by diet, environment, host genetics and exposure to antimicrobial compounds^8^. Clinical evidence suggests that mycobiome communities are considerably less stable than bacterial communities in the same individuals across time. In addition, it remains uncertain whether many rare intestinal fungi are active gut colonizers or simply transiting the intestine after being ingested^9^. Despite this uncertainty, emerging preclinical research has identified distinct fungal community compositions associated with murine metabolic phenotypes and influenced by host factors.

The host environment is a key driver of mycobiome colonization^10–12^. Mims et al. compared the mycobiome of C57BL/6 mice from four commercial vendors upon arrival at their institution and following 8 weeks of dietary intervention^10^. At baseline, distinct differences in fungal communities were detected between mice from different vendors, which were maintained through the 8-week experiment^10^. The relative abundances of specific fungal genera were correlated with adiposity and endocrine markers (that is, triglycerides, insulin, glucagon, leptin and ghrelin), and contributed to vendor-specific metabolic responses to the experimental diets^10^. Similarly, Doron et al. compared the mycobiome structure and the immune responses of commercial C57BL/6 mice with those of animal colonies maintained at their institution^11^. Although they found differential fungal community compositions between colonies, colonies of both origins mounted a T helper type 17 cells (T_H_17) immune response to *Candida albicans* colonization, demonstrating that key host immune pathways were preserved despite differential mycobiome compositions. Furthermore, Bendova et al. analyzed the bacterial and fungal microbial communities between wild-caught *Mus spicilegus* and captive *Mus musculus* species^12^. They demonstrated that the mycobiome of *M.* *spicilegus* is significantly more diverse than that of laboratory *M.* *musculus* and posited that human maintenance of *M.* *musculus* colony lines skewed endogenous fungal and bacterial communities compared with their wild counterparts^12^. These are just a few studies that demonstrated the impact of the environment on the gut mycobiome composition and that specific fungal taxa, such as *Candida* and *Aspergillus*, contribute to host metabolic and immunological homeostasis.

While host environment is a potent modulator of mycobiome community composition, host genetics seems to be another important driver in fungal colonization as demonstrated by Gupta et al.^13^. They tested the relationships between gut fungal taxa and host genetics in the context of three different dietary interventions in an intercross mouse line. Four mouse strains (MRL/MpJ, NZM2410/J, BXD2/TyJ and Cast/Ei) were purchased and bred for 20 generations to produce an experimental cohort of 591 animals^13^. After this complex breeding process, animals underwent a 5-month dietary intervention with groups receiving a high-fat Western diet or a calorie-restricted diet^13^. Quantitative trait loci mapping allowed for an analysis of the role of host genetics on fungal microbiome composition^13^. Gupta and colleagues found that the interaction between host genetics and diet explained 33% of gut fungal phenotypic variation, and they identified several candidate genes related to chronic metabolic and inflammatory diseases (that is, *Taf4b*, *Tmc8*, *Wdr11* and *Grk5*) that may modulate mycobiome composition^13^. This study demonstrated the complex interplay between host genetics and the adaptability of the gut mycobiome across several clinically relevant dietary contexts.

Finally, gut ecology is directly modulated by exposure to antimicrobial compounds. The antibiotic treatments administered for bacterial infection deplete gut bacterial communities, while the gut mycobiome is known to be more resilient^14,15^. A 2013 study by Dollive et al. examined the effects of short-term or continuous antibiotic use on the mouse microbiome over 76 days^15^. The investigators observed that antibiotic-induced disruption of bacterial communities exacerbated intestinal fungal colonization, with increased abundance of *Candida* both during and after antibiotics exposure^15^. Thus, these findings reveal a dynamic and competitive interplay between the microbial and fungal compartments of the gut microbiome, disruptions of which impact host disease susceptibility.

Together, the host environment, genetics and the exposure to antimicrobial compounds have wide-ranging and interrelated implications on the composition of the gut mycobiome. These influences have also important roles in the colonization of key fungi that drive metabolic and immunological health and disease. In the following sections, we will review the findings from different rodent studies that have demonstrated a link between fungal communities and key disease states.

## The impact of the intestinal mycobiome on disease

### Inflammatory bowel disease

**Fig. 1:**
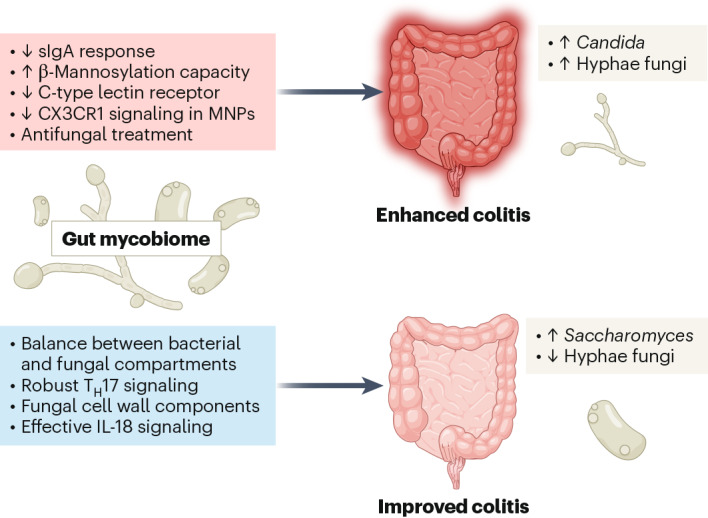
Overview of the impact of the gut mycobiome on colonic health. The effects of the mycobiome on colonic health are mediated through the shaping of the fungal community composition, mucosal immune surveillance, signaling and response. Gut mycobiome imbalance can negatively impact gut health by reducing sIgA response, impairing C-type lectin receptor and CX3CR1 signaling in MNPs, and increasing β-mannosylation capacity. In some conditions, the use of antifungal treatment can negatively affect colonic health. Conversely, gut mycobiome homeostasis can impact gut health positively by promoting robust T_H_17 signaling (important for host defense and barrier integrity), improving beneficial fungal growth and inducing effective IL-18 signaling.

Given that the gut mycobiome has a direct interaction with the intestinal tissue, the role of the gut mycobiome on gut diseases has become a key area of research. Inflammatory bowel diseases (IBD) are proinflammatory conditions of the intestinal lining, most diagnosed as either Crohn’s disease or ulcerative colitis^16^. Crohn’s disease can occur throughout the length of the gastrointestinal tract and is characterized by mucosal thickening, stenosis, restructuring and fibrosis, while ulcerative colitis is limited to epithelial ulceration of the colonic mucosa^16^. Mouse models have been particularly useful in investigating the influence of the mycobiome on the pathophysiology of these diseases. Intriguingly, as we will summarize below, the evidence suggests that the presence of key fungi in the gut can either contribute to or protect against the risk of IBD (Fig. 1).

#### Effect of colitis on murine intestinal mycobiome and pathogenicity

Several studies have explored the relationships between mycobiome community composition, the presence of specific fungi and colitis pathogenesis. Notably, Wheeler et al. investigated the impact of fungal dysbiosis induced by fluconazole treatment in chemically induced and T cell transfer colitis models^17^. They demonstrated that antifungal treatment restructured the fungal community and increased the severity of colitis in both models. They implicated key fungi (for example, *Candida*, *Aspergillus amstelodami*, *Wallemia sebi* and *Epicoccum nigrum*) as modulators of IBD severity in mice^17^; more specifically they showed that *Candida* spp. was reduced, while *Aspergillus*, *Wallemia* and *Epicoccum* spp. were increased after fluconazole treatment. However, these fungi found to expand during antifungal treatment were not sufficient to induce disease in models utilizing dextran sodium sulfate (DSS), suggesting additional mechanisms of antifungal-induced disease pathogenesis^17^.

Similarly, Qiu et al. investigated fungal intestinal localization in DSS-induced colitis mouse models and found increased abundance of *Penicillium*, *Wickerhamomyces*, *Alternaria* and *Candida*, and decreased abundance of *Cryptococcus, Phialemonium* and *Wallemia* in the guts of DSS colitis mice compared with nontreated controls^18^. Depletion of fungi using antifungal treatment worsened DSS-induced colitis in mice and decreased butyrate-producing bacteria compared with nontreated controls^18^. Mice with chronic recurrent DSS colitis presented with fungal translocation into the colonic mucosa, mesenteric lymph nodes and spleen^18^. The researchers concluded that the mycobiome has a critical antagonistic role in balancing bacterial microbiota to maintain intestinal homeostasis, and also demonstrated the capacity of fungi to translocate harmfully in a chronic colitis scenario^18^.

Microorganism translocation from the intestinal tract is generally a negative result of colitis and induces a strong host immunological response^19^. A functional intestinal microbiome protects intestinal tissue against inflammatory disease and infection and also enhances immune cell responsiveness to pathogens^20^. While this protective activity is recognized for commensal bacteria, Jiang et al. investigated whether commensal fungi may also protect mucosal tissue by interacting with immune cells. The researchers devised three experimental groups: one group of mice that received DSS to induce colitis, the second group that received broad-spectrum antibiotic (ABX) and a third group that received both DSS and ABX. After treatment, animals were inoculated with monocultures of *C.* *albicans* or *Saccharomyces cerevisiae*^20^. Both monocultures established fungal colonies post-antibiotic treatment and ABX mice inoculated with *C.* *albicans* gained weight comparable to ABX mice^20^. Furthermore, the monocultures protected against DSS colitis-induced mortality compared with the fungi-free ABX mice^20^. Both monoculture-treated ABX groups exhibited CD8^+^ T cell counts reflective of the no antibiotic-treated control groups and protection against colonic shortening^20^. Finally, this study demonstrated that fungi-specific cell wall glycoproteins (that is, mannans) conferred protection against colitis in the absence of fungal inoculation via TLR4 signaling^20^. These results demonstrated that commensal fungi might functionally maintain immune cell response to colitis in the absence of commensal bacteria^20^.

While commensal species can confer protection against colitis, once colitis is established, the translocation of microorganisms can cause severe infections^21^. As such, Jawhara et al., investigated the higher mortality rate seen in *Candida glabrata* infections compared with *C.* *albicans* infections. *C.* *glabrata* is known to be resistant to antifungal treatments, making infection more difficult to remediate^22^. Both species show β-mannosylation capacity, which contributes to cellular adhesion, gut colonization and host immune activation^22^. Jawhara and colleagues generated β-mannosyltransferase-deficient strains of *C.* *glabrata* and demonstrated decreased colonization and improved host phenotype (that is, colonic inflammation, weight loss and mortality) in DSS mice treated with the deficient *C.* *glabrata* compared with DSS mice treated with the wild-type *C.* *glabrata* strain, highlighting that mannosyltransferase activity is a key functional aspect of *Candida* pathogenicity in colitis^22^.

These insights provide evidence for fungal dysbiosis as a key element of the etiology and pathophysiology of IBDs, further demonstrating the complex relationships between the bacterial and fungal compartments of the intestinal microbiome. The contribution of the mycobiome to host immunological function during colitis calls for further study into host−mycobiota signaling pathways that may reveal therapeutic opportunities.

#### Fungal pathways affecting colitis susceptibility and severity

Several fungal recognition targets in the murine colon have been identified as mediators of colitis severity^23^. Deficiencies in C-type lectin receptors (CLRs) that act as fungal recognition domains and activate the intracellular Caspase recruitment domain 9 (CARD9) are known to increase colitis severity in mice^24^. Dectin-1 is a CLR expressed in myeloid cells with specificity for β-1,3-glucans that compose fungal cell walls and are pervasive across fungal species^25^. In 2012, Iliev et al. determined that knocking out the gene for dectin-1 in mice (*Clec7a*^*−/−*^mice) increased susceptibility to DSS colitis independent of commensal bacteria^24^. Increased susceptibility to DSS-induced colitis was not transferred to wild-type mice with fecal microbiota transplant (FMT) from *Clec7a*^−/−^ mice^24^. This finding indicated that host genotype and not differential abundance of fungi drives colitis severity, further rooting the importance of dectin-1 in mediating host immunological homeostasis^24^.

Dectin-1 triggers the CARD9 pathway, which induces T_H_17 host defense response^26^. Malik et al. showed that this signaling axis requires gut commensal fungi for the prevention of colitis and colorectal cancer^26^. Mice lacking a functional SYK−CARD9 axis exhibited defective inflammasome activation and reduced IL-18 secretion due to decreased caspase-1 activation in DSS-induced colitis^26^. Supplementation of IL-18 reduced colitis-induced inflammation and protected against the physiological deficits associated with colitis^26^. Thus, the protective effect of the mycobiome was dependent upon the SYK−CARD9 axis and inflammasome aggregation, promoting IL-18 maturation and an antitumorigenic T cell response^26^.

Myeloid cells are key immune cells that contain the SYK pathway and are critical for host−mycobiome homeostasis^26^. CX3CR1^+^ mononuclear phagocytes (CX3CR1^+^ MNPs) are a type of local myeloid cell that are found in the intestinal lamina propria and prime T_H_17 cells to trigger antifungal responses^27^. To investigate this specific immune pathway in vivo, Leonardi et al. colonized mice with *C.* *albicans* and evaluated the changes in the surface expression of costimulatory molecules (that is, CD40 and CD86) in CX3CR1^+^ MNPs^27^. They found that transcripts encoding CLRs were highly present in CX3CR1^+^ MNPs and, via in vivo confocal microscopy, found that 80% of CX3CR1^+^ MNPs were recognizing and intaking *Candida*^27^. Furthermore, in a CX3CR1^+^ MNP depletion model, they confirmed a decrease in T_H_17 cell response and exacerbation of DSS-induced colitis after *Candida* colonization^27^. The T_H_17 response was dependent on an intact SYK signaling within CX3CR1^+^ MNPs, revealing the role of CX3CR1^+^ MNPs in maintaining homeostasis between the immune system and the colonic mycobiome in colitis^27^.

Much of the cited preclinical literature in this section has identified *Candida* as a key genus present in colitis. Indeed, *Candida* is a commensal fungus that has been frequently observed to colonize patients with ulcerative colitis^28^. More specifically, the species *C.* *albicans* has been found in 91% of cases^29^. In rats, *Candida* colonization delayed healing in chemically induced ulcerative colitis, as indicated by decreased colonic blood flow, and elevated circulating levels of IL-1β and TNF^29^. These parameters were improved with antifungal treatments, demonstrating that *Candida* colonization was contributing to disease severity^29^. On the other hand, in a mouse model of DSS colitis, *S.* *cerevisiae* var. *boulardii* was shown to compete with *C.* *albicans* and reduce inflammation^30^. On the basis of these findings, Jawhara et al., investigated the independent effects of several *S.* *cerevisiae* strains, as well as mannoproteins and β-glucans derived from *S.* *cerevisiae* and *C.* *albicans* in a curative model of DSS colitis in BALB/c mice^31^. The results demonstrated that *S.* *cerevisiae* had a strain-dependent effect on colitis severity, with the strain most closely resembling var. *boulardii* reversing all aspects of colitis (for example, histological damage, diarrhea and TNF expression)^31^. In addition, β-glucans, regardless of their source, prevented *C.* *albicans* colonization and intestinal inflammation^31^.

However, Chiaro et al. found that *S.* *cerevisiae* exacerbated intestinal disease and increased barrier dysfunction in a mouse model of colitis^28^. They showed that *S.* *cerevisiae* colonization downregulated the expression of epithelial tight junction genes and increased uric acid production in germ-free mice. Uric acid is the cause of uric acid nephrolithiasis, a condition more likely to be present in patients with IBD^28^; however, the mechanism is not fully clear. Taken together, these studies (refs. ^28,31^) demonstrate the complex nature of the gut mycobiome and suggest that strain-level differences can have potent effects on host physiology. Furthermore, Jawhara et al. suggested that multivalent binding is necessary for strong inflammatory response as isolated fungal cell wall constituents had protective effects^31^.

*C.* *albicans* is a dimorphic fungus, capable of altering its cellular morphology between yeast and hyphae states in response to the host environment^32^. Doron et al. demonstrated that *C.* *albicans* colonization drives IgA secretion and that secretory IgA (sIgA) preferentially binds the hyphae fungi, thereby limiting virulence and promoting commensalism^32^. This sIgA response is disrupted in patients with IBD, who exhibit decreased sIgA and increased fungal hyphae, which could promote disease severity^32^.

Botschuijver et al. reported two studies investigating the role of the gut mycobiome in IBD. In the first study, the researchers analyzed the role of the gut fungal community in a rat model of stress-induced visceral hypersensitivity^33^. This model relies upon maternal separation that imitates early life stressors that are associated with the development of IBD in adulthood^33^. The experiments demonstrated that fungal dysbiosis in rats that had been separated from their mothers promoted visceral hypersensitivity, also suggesting that therapy with antifungals, soluble β-glucans or a SKY inhibitor can reduce IBD-related visceral hypersensitivity^33^. In the second hypersensitivity study, the researchers used nonhandled and maternally separated rats and treated them with menthacarin (a combination of essential oils from peppermint and caraway). The results indicated that menthacarin could reverse visceral hypersensitivity through gut mycobiome modulation^34^.

Overall, colitis in the context of IBD has a disruptive effect on mycobiome homeostasis leading to the outgrowth of key genera and exacerbating the pathogenesis of the disease. While the causative impact of mycobiome community composition on the etiology of IBD is still under investigation, it is apparent that fungal pattern recognition receptors and downstream immunological responses have a key role in exacerbating or ameliorating the condition. In addition, these studies have collectively demonstrated the potential probiotic properties of several fungal genera, and that isolated fungi-derived proteins may provide therapeutic benefits in colitis. These new insights warrant further clinical investigation.

### Metabolic diseases

Alterations in the gut mycobiome can have wide-ranging effects on nutrient absorption, impacting host metabolism beyond the intestinal tract. Numerous studies have now linked the gut microbiome to metabolic diseases including diabetes, obesity and liver disease^35^. A primary insult in such diseases is dietary intake, specifically a high-fat or industrialized diet. In one of the first murine reports, Heisel et al. analyzed the effect of a high-fat diet on the gut mycobiome and showed that a high-fat diet increased *Candida* compared with chow controls^35^. Further, Sun et al. determined that fungi sensitive to amphotericin B and fluconazole were involved in obesity pathogenesis, as mice treated with these antifungal agents did not exhibit weight gain in a diet-induced obesity model^36^. *Candida parapsilosis* and *Naganishia globose* were enriched in control animals on a high-fat diet, and treatment with the isolated *C.* *parapsilosis* resulted in elevated intestinal free fatty acids in fungi-free mice^36^. In diet studies, *C.* *albicans* has been shown to function in a more mutualistic and diet-dependent manner, moderating metabolic dynamics in the gut^37^. Peroumal et al. demonstrated that *C.* *albicans* differentially induced hormone response between a high-fat diet mice and controls^37^. High-fat diet fed animals exhibited elevated insulin, leptin, resistin and abdominal adiposity compared with the normal-diet controls^37^. When additionally supplemented with *C.* *albicans*, the differences between diet groups were largely ablated, with *Candida*-treated high-fat diet fed animals maintaining reduced glucagon-like peptide 1 (GLP1) as the only notable difference. As such, the effects of the *Candida* genus are context dependent and may be a useful target in the treatment of obesity and metabolic disorders.

Type 2 diabetes mellitus (T2DM) is widely known to be associated with drastic shifts in gut microbiota composition^38^; however, the impact of diabetes on gut fungi is largely unexplored. Metformin is a biguanide drug commonly prescribed in T2DM treatment that improves peripheral insulin sensitivity, reduces intestinal glucose absorption and inhibits hepatic gluconeogenesis. In one study, C57BL/6 mice treated with metformin demonstrated a gut enrichment in *Fusarium*, *Thermothielavioides*, *Cryptococcus* and *Tetrapisispora*, together with a decrease in *Saccharomycetales* compared with placebo-treated mice^39^. Other studies have demonstrated that the rodent mycobiome composition is drastically shifted in T2DM^40,41^. For example, Padakandla et al. noted an increased abundance of opportunistic pathogenic fungi, such as *Aspergillus*, *Candida* and *Isaatchenkia*, which can potentially increase proinflammatory responses in diabetic rats compared with controls^41^. They also showed that animals presenting with a more severe diabetic phenotype (that is, diabetic retinopathy) developed a mycobiome composition distinct from the diabetic animals without retinopathy^41^. While GLP1 agonists have gained popularity in the pharmaceutical treatment of T2DM and obesity, previous work has demonstrated that the microbiota is involved in GLP1 secretion^42,43^ and treatment efficacy^44^. However, the involvement of the gut fungal compartment in the mechanism of action of GLP1 remains an open question and warrants further investigation.

According to past findings, the intestinal mycobiome is related to alcoholic and nonalcoholic liver disease outcomes. While ethanol consumption is known to contribute to gut microbiota overgrowth^45^, the role of mycobiota is less clear. Several studies have demonstrated that antifungal treatment can ameliorate alcohol-induced liver injury in rodents^46,47^. Yang et al. explored the contribution of gut mycobiome immune signaling on the development of alcoholic liver disease in mice^46^. They showed that ethanol administration increased gut fungal abundance and diversity, characterized by elevated *Humicola*, *Fusarium*, *Aspergillus* and decreased *Candida* species. Antifungal treatment decreased liver injury, β-glucan translocation and hepatic immune cell infiltration in the animals on the ethanol diet^46^. The study also revealed that the effect of fungi on the pathophysiology of alcoholic liver disease was mediated by dectin-1 signaling in Kupffer cells^46^. Sun et al. showed that water-insoluble polysaccharides derived from the plant fungus *Wolfiporia cocos* ameliorated ethanol-induced gut mycobiota dysbiosis and lipopolysaccharide absorption, and improved markers of hepatic stress in mice^47^. This study also revealed that *Meyerozyma* overgrowth in the ethanol-treated animals was directly linked to the severity of alcoholic hepatic steatosis^47^. Nonalcoholic fatty liver disease and steatohepatitis have also been tied to gut fungal abundance^48^. Demir et al. found distinct mycobiome signatures between human patients with nonalcoholic fatty liver disease and nonalcoholic steatohepatitis^48^. They also showed that in fecal microbiome-humanized germ-free mice, antifungal treatment protected against Western diet-induced lipid deposition, inflammation and upregulation of transcripts involved in fibrosis^48^.

Taken together, these studies demonstrate the impact of the mycobiome beyond the local gut environment, highlighting important links between metabolic disease (that is, obesity, diabetes and liver diseases) and alterations in the gut mycobiome. Most of these studies used antifungal treatment to deplete the gut mycobiome and/or isolation and administration of single fungal species. While this method is useful for probiotic development and identification of targeted treatments, the gut biome is complex and more work is needed to further uncover the effects of the different gut biome compartments and their interactions in the development of these diseases.

### Cancer

Cancer is a highly heterogeneous disease with varying etiology, pathophysiology and tumor microenvironments depending on the body site^49^. In patients, distinct fungal signatures have been detected in the tumor microenvironment depending on cancer type and location^50^. While the literature exploring the implications of the mycobiome on cancer is limited, animal models have unveiled relationships between gut fungal communities and tumor progression^51^. Antifungal treatment has been shown to improve radiation therapy in mouse models of breast cancer and melanoma^52^ and to block lung cancer progression^53,54^. Much of the work investigating the effects of the gut mycobiome on cancer, unsurprisingly, focuses on colorectal cancers; however, research on cancers of other metabolic tissues (that is, pancreas or liver) is also developing.

In azoxymethane DSS-induced models of colorectal cancer, *C.* *tropicalis* has been shown to facilitate the immunosuppressive function of myeloid derived suppressor cells (MDSCs)^55,56^. Dectin-1 deficiency decreased the infiltration of MDSCs and the expression of prostaglandin, while enhancing the expression of tumor inhibitory IL-22-binding protein (IL-22BP) in mouse colonic tumors^57^. Dectin-1 is expressed ubiquitously in the host immune cells^26^, and activates the SYK−CARD9 signaling axis, which promotes gut fungi-mediated inflammasome activation^26^. CARD9 expression is increased in immune cells during colonic tumorigenesis^26^. Compared with controls, CARD9-deficient mice lost more body weight, had increased tumor burden, greater colonic inflammation and epithelial hyperplasia^26^. These findings were replicated with antifungal treatment and reversed with IL-18 supplementation, suggesting that fungal induction of the inflammasome via the CARD9 pathway in CD8^+^ T cells is protective against colon tumorigenesis^26^. Taken together, these findings indicate that canonical CLR signaling pathways may have differential downstream effects in specific immune cell types involved in tumorigenesis, notably in MDSCs and T cells, where dectin-1 drives tumorigenesis and CARD9 prevents tumorigenesis in a somewhat oppositional relationship in the colorectal tumor microenvironment^58^.

Aykut et al. examined the role of the gut mycobiome in pancreatic ductal adenocarcinoma (PDA) using the p58/KC mouse model, which develops PDA spontaneously^51^. They showed that *Malassezia* spp. translocated from the intestine to the pancreas, which potentiated PDA tumorigenesis^51^. Fungal ablation with amphotericin B was protective against oncogenesis both in vivo and in vitro, and repopulation of the gut specifically with *Malassezia-*accelerated oncogenesis^51^.

Few studies have investigated the role of the mycobiome in hepatic cancer types. Studies in the past 5 years have demonstrated that patients with hepatocellular carcinoma harbor a gut mycobiome less diverse than patients with liver cirrhosis, despite an overrepresentation of *C.* *albicans*^59–61^. In the Hepa 1-6 syngeneic mouse model, in which mice were pretreated with antibiotics, *C.* *albicans* gavage resulted in greater tumor burden and induction of *Nlrp6* expression compared with control mice administered PBS^59^. *Nlrp6* knockout ameliorated the deleterious effect of *C.* *albicans*, suggesting that intestinal NACHT, LRR and PYD domains-containing protein 6 (NLRP6) mediates the effect of mycobiota on tumor growth in mouse hepatocellular carcinoma. By contrast, *S.* *cerevisiae* gavage decreased inflammation and tumor incidence in a mouse model of DEN^+^CCl_4_-induced primary liver disease^60^.

Collectively, research in the cancer area points to a site-specific effect of distinct fungi, both at the genus and species level, on tumorigenesis, radiation therapy efficacy and immune system activation. While the specific taxa may differ across cancer type, fungal CLRs in tumor immune cells seem poised to mediate the effects of the local and systemic mycobiome on cancer progression and metastasis.

### Neurodegenerative disease and stroke

The influence of gut microbe dysbiosis on central nervous system function and the development of neurodegenerative disease is another emerging area of research^62^. Recently, studies exploring the gut−microbiota−brain axis have notably investigated the contribution of mycobiome disruption in neurodegenerative diseases such as Alzheimer’s disease. Characterization of gut fungal communities in the 3xTg-Alzheimer’s disease mouse model revealed an enrichment of the *Dipodascaceae* family relative to wild-type mice^63^. This yeast family is also reported to be abundant in obese mouse models, suggesting that the enrichment of this fungi family might be related to proinflammatory imbalances^63^.

Ye et al. investigated the effect of *Saccharomyces boulardii* on the regulation of microglia-induced neuroinflammation in Alzheimer’s disease using APP/PS1 mice^64^. Treatment of Alzheimer’s disease mice with *S.* *boulardii* improved gut-barrier integrity and synaptic plasticity, and decreased β-amyloid deposition, microglia-mediated neural inflammation and toll-like receptor expression^64^. The beneficial effects were ablated with antifungal treatment, suggesting that *S.* *boulardii* may have an acute role in moderating neuroinflammation via toll-like receptors and gut−brain axis signaling.

### Allergic airway disease

Just as studies have implicated the mycobiome in immune-mediated IBDs, studies have shown that the fungal compartment of the gut microbiome is a key factor in the pathogenesis of allergic airway disease^17^. Allergic airway disease is defined by a specific inflammation pattern that leads to the recruitment of eosinophils, type 2 macrophages, innate lymphoid cells type 2, IgE-secreting B cells and T helper type 2 (T_H_2) cells^65^. Allergic airway diseases are also characterized by the prominent production of specific cytokines, such as IL-4, IL-5 and IL-13 (ref. ^66^). In humans, gut microbiota dysbiosis has been associated with elevated immune responses in the allergic response^67^. Recently, several mouse models have been used to identify the underpinning mechanisms and the potential contribution of the gut mycobiome in allergic airway diseases.

*Candida* enrichment in the airways has been positively associated with airway immune response to allergic insults^68^. Evidence suggests that airway immune responses are exacerbated with antibiotic exposure^14^. As discussed previously, *Candida* species in the gut increase in relative abundance during host antibiotic treatment^15^. Kim et al. found that mice pretreated with antibiotics developed elevated airway immune response when challenged with papain^14^. In the absence of gut bacteria, the level of fungal overgrowth in the gut correlated with severity of airway inflammation; prostaglandin synthesis by *C.* *parapsilosis* was specifically implicated in this process^14^. Antibiotic exposure or absence of gut bacteria have consistently exacerbated allergic airway disease across several methods of induction^14,17,69–71^. Similarly, Noverr et al. observed that antibiotic-induced *C.* *albicans* enrichment triggered airway allergy in mice through the priming of CD4^+^ T cells and IL-13 induction that perpetuates T_H_2 responses^70^. CX3CR1^+^ MNPs, present in the airway, are key immune cells moderating the immune response to the mycobiome, specifically via T_H_17 signaling^71,72^. Li et al. showed that while airway CX3CR1^+^ MNP frequencies did not change during house dust mite-induced allergic airway disease, SYK signaling in intestinal CX3CR1^+^ MNPs was necessary to perpetuate T_H_2 signaling in the airway and moderate the immune tone^71^. Wheeler et al. hypothesized that antifungal agents may alleviate immune activation in a mouse model of allergic airway disease; however, they observed exacerbated allergic airway disease after treatment^17^. This response was driven by the antifungal-induced expansion of three resistant gut fungi: *Aspergillus amstelodami*, *Epicoccum nigrum* and *Wallemia sebi*^17^.

These findings have also been replicated in models of bronchopulmonary dysplasia. Willis et al. tested whether the gut mycobiome profile could promote lung injury in bronchopulmonary dysplasia (BPD) in mice. They administered ABX to C57BL6/J female mice to disrupt the gut microbiome, before performing FMT with samples from infants with BPD and post-prematurity respiratory disease. Then, they bred the mice, and administered a second FMT to the pups, using the same donor samples administered to the dams. After FMT, neonatal mice were exposed to hyperoxia to induce an analogous BPD lung injury. The findings showed an association between the fungal intestinal microbiota composition and the severity of the lung injury, demonstrating a previously overlooked gut-lung axis for fungal colonizers^73^.

## Conclusions and future directions

Over the past two decades, microbiome research has understandably focused on intestinal bacterial community composition and its functional impact on host health. While the bacterial compartment comprises 99% of the gut microbiome, recent advances in sequencing and computational technology have uncovered the contribution of the mycobiome compartment to host health. These insights have demonstrated the importance of homeostasis in the dynamic interplay between the bacterial and fungal compartments. Even subtle perturbations in the community compositions can have a great impact on microbiome functionality, with direct consequences on host health and disease pathogenesis. Future research should continue to investigate the mycobiome using preclinical rodent models, including wildings and other dirty mouse models. These models will help us to further understand the delicate microbe-microbe and host-microbe dynamics that contribute to health and disease and that could be leveraged as treatments to optimize human health.
